# Turning Titanium Alloy, Grade 5 ELI, With the Implementation of High Pressure Coolant

**DOI:** 10.3390/ma12050768

**Published:** 2019-03-06

**Authors:** Bogdan Słodki, Wojciech Zębala, Grzegorz Struzikiewicz

**Affiliations:** Production Engineering Institute of the Mechanical Faculty, Cracow University of Technology, Al. Jana Pawła II 37, 31-155 Kraków, Poland; zebala@mech.pk.edu.pl (W.Z.); struzikiewicz@mech.pk.edu.pl (G.S.)

**Keywords:** turning, Titanium alloy, high pressure coolant, cutting forces, chips form

## Abstract

In the machining of difficult-to-cut alloys, such as titanium-based alloys, the delivery of a cutting fluid with high pressure can increase machining efficiency and improve process stability through more efficient chip breaking and removing. Proper selection of machining conditions can increase the productivity of the process while minimizing production costs. To present the influence of cutting fluid pressure and chip breaker geometry on the chip breaking process for various chip cross-sections Grade 5 ELI titanium alloy turning tests were carried out using carbide tools, H13A grade, with a -SF chip breaker geometry under the cutting fluid pressure of 70 bar. Measurements of the total cutting force components for different cutting speeds, feeds, and cutting depth in finishing turning were carried out. The analysis of the obtained chips forms and the application area of the chip breaker have been presented. It was proved that for small depth of cut (leading to small chip cross-section) the cutting fluid pressure is the main cause of the chip breakage, since the insert chip breaker does not work. On the other hand, for bigger depths of cut where the chip breaker goes in action, the cutting fluid pressure only supports this process. For medium values of depths of cut the strength of chip is high enough so that the pressure of the cutting fluid cannot cause chip breaking. A chip groove is not filled completely so the chip breaker cannot play its role.

## 1. Introduction

A properly controlled machining process allows increasing productivity and reducing the costs of energy, materials, and tools [1,2,3]. Obtaining high efficiency and quality of difficult-to-cut materials machining with minimal manufacturing costs requires thorough research and analysis. The hard material group includes materials characterized by high mechanical properties, low thermal conductivity, and high chemical reactivity. These include heat-resistant steels, nickel- and cobalt-based super alloys and titanium alloys. Due to their properties, titanium alloys have found wide application in many branches of industry, such as in chemical and petrochemical industries, shipbuilding (excellent corrosion resistance), medicine, and dentistry for surgical and prosthetic implants [4]. The high strength-to-weight ratio of titanium alloys (titanium density is ~60% relative to nickel or cobalt-based super alloys) predestines this material for the construction of components used in the automotive, aerospace, and automotive industries [5].

During machining, the main problems are high temperature and concentration of stresses on the cutting edge, which causes the accelerated tool wear [6,7,8]. This is partly due to the poor thermal conductivity of titanium alloys. Da Silva et al. [6] showed that adhesion and attrition are dominant wear mechanisms in the machining of a Ti–6Al–4V alloy with PCD (Polycrystalline Diamond) tools with a high pressure coolant. Similar conclusions can be drawn from research conveyed by Ren et al. [8] regarding machining with TiAlN-coated cemented carbide tools with chip groove. In turn, tool life improvement can be obtained from machining with cooling fluids. Hong et al. [7] presented that cryogenic machining approach is better than all known metal cutting methods even such as ultrahigh-pressure water jet-assisted machining. A significant part of the heat generated during the cutting process (approximately 80%) is supplied and transferred by the cutting tool [9,10]. In order to improve the machinability of titanium alloys (and other hard-to-cut materials), various techniques of cooling and lubrication of the cutting zone have been developed, such as MQL (Minimum Quantity Lubrication), HPC (High Pressure Cooling) [10,11,12,13], and cryogenic machining [7,12]. A review of cryogenic cooling methods was described in details in Sulaiman et al. [14] e.g., CO_2_ and liquid nitrogen. One of the most commonly used techniques in the manufacturing industry is the system-enabling feeding of cutting liquid to the cutting zone under high pressure (from 50 to 355 and even 1000 bar) [1]. The use of HPC during the machining of titanium and nickel alloys enables the increase of machining efficiency, control of shape and chip form by supporting the chip breaking process, increasing the efficiency of chip removal outside the machining zone, and a significant temperature reduction in the cutting zone, which extends the service life of the cutting tool (5–15 times) [6,9,15,16]. Da Silva and Palanisamy et al. confirmed that high pressure coolant flow traverses the surface faster, significantly lowering the film boiling action of the coolant, penetrating deep into the cutting area, and achieving high chip breakability through increased chip up-curl. In case of machining Inconel 718 (Ezugwu, Polvorosa et al.), especially at higher speed conditions, with coated carbide tools under high-pressure coolant supplies tool life can be improved by up to 7-fold. Compared to the conventional cooling, HPC machining can increase production through higher cutting speeds [6,10].

The area of research described in the literature regarding the machining of titanium alloys mainly involves the influence of cutting conditions (most commonly cutting speeds and feed and depth of cut) on the quality of machined surface, chip creation and breaking, determination of cutting force values, temperature of the cutting zone, among others, for hard-to-machine materials [1], milling [17], and turning [18] processes. Palanisamy et al. [15] conveyed research concerning turning a Ti6Al4V alloy. A series of experiments were carried out with different cutting parameters and cutting fluid pressure levels for machining with uncoated carbide inserts. Studies have shown that the use of high pressure ensures improved chip breaking and evacuation due to its mechanical action. They also found that HPC machining increases cutting edge durability almost three times compared to conventional cooling [6,7]. Çolak [11,19] investigated the machinability of Inconel 718 and titanium alloy in conventional and high-pressure cooling conditions at different cutting speeds, feeds, and cutting depths with the help of a sintered carbide tool (Ti, Al) N + TiN). He found that feeding high pressure coolant into the cutting zone reduces the values of the cutting force components, ensures the desired chip breakage, and causes less wear of the cutting tool. Da Silva et al. [6] analyzed the tool wear mechanism. Tests concerned machining titanium alloy at high speed machining conditions. It turned out that tool life decreased with increasing cutting speed despite the cooling system. Significant improvement in productivity was achieved when machining with high pressure coolant supply. Cutting fluid applied from overhead tends to boil and vaporize due to the high temperature at the primary shear zone. However, high pressure causes the fluid to access the sliding zone lowering friction and temperature. The high pressure coolant jet tends to lift the chip resulting in a reduction in the tool–chip contact length and the chip curl radius (Ezugwu et al., 1990). Reduction in the chip curl radius is a prerequisite for efficient chip breaking. Segmented chips were generated when machining with HPC, while continuous chips were generated during machining with conventional coolant flow. In turn, Kamiński and Alvelid [18] analyzed the temperature in the cutting zone under the HPC conditions. The authors say that the decrease in temperature in conventional cooling in comparison with dry machining is ~15% while that decrease of the temperature when HPC is ~40–45%. This phenomenon takes place when the pressure is between 20 and 70 MPa. Water flow (l/min) does not affect the temperature. Higher coolant pressure does not lower the temperature. Additionally, high coolant pressure “strongly influences the chip formation process and thus chips breaking.”

A number of described studies concerned the optimization of the titanium alloys machining using various methods and mathematical models. For example, Cus et al. [20] demonstrated the usefulness of genetic algorithms (GA) for online optimization of cutting data in the milling process. Raja and Baskar [21] conducted research on the optimization of machining parameters due to the desired surface roughness. Heron [22], Safari [17], and other authors [2,4,17,23] developed an optimization model for the turning process taking into account factors affecting machining, such as cutting force (*F_c_*), tool life (*T*), surface roughness (*Ra*), material removal rate, and chip breakability. Surface roughness and chip breakability were selected as optimization criteria due to their importance for the finishing turning process. Several studies that used the same approach can be found in the literature [3,8].

Due to the problems described above, for obtaining an effective machining of titanium alloys, a series of Ti6Al4V ELI alloy tests were carried out under the conditions of applying cutting fluid at increased pressure. Ti6Al4V ELI (Extra Low Interstitials) is very similar to Ti6Al4V, except for the reduction in oxygen, nitrogen, carbon, and iron content. This ensures better ductility and fracture toughness, with a certain reduction in strength. The improve mechanical properties are the dominant reason for the selection this material for the reliable applications, e.g., in dentistry and medicine for heavily loaded implants [5,24,25,26]. The experiments were designed based on the Taguchi method [24] at different cutting speeds (*v_c_*), feeds (*f*), and cutting depths (*a_p_*). During the experiments, cutting forces (*F_c_*, *F_p_*, and *F_f_*) were recorded and chip analysis was executed at the constant value of cutting fluid pressure.

## 2. Materials and Methods 

The chemical composition of the workpiece is presented in Table 1. A titanium round bar, hot-forged, annealed, and peeled was used as the workpiece. Its tensile strength was 902 MPa and its Rockwell hardness was 29 HRC. The turning process was analyzed in the conditions of increased cutting fluid pressure. Cutting inserts CNMG120404-SF (*r_ε_* = 0.4 mm) of the H13A grade, -SF chip breaker, and the tool holder PCLNR2020K12HP, from Sandvik Coromant (Sandviken, Sweden), were used in the cutting tests. The workpiece as a shaft with diameter *Dc* = 50 mm was machined. The cooling liquid was a 10% Blasocut 2000 universal emulsion from Blaser (Rüegsau, Switzerland). 

During the tests, measurements of the components of the total cutting force and microscopic measurements of the chip dimensions and the rake surface of the cutting insert were made. In order to register and analyze the components of the cutting forces, a measuring system based on the 9257B dynamometer and the Kistler 5070B amplifier was used—produced by Kistler Company (Winterthur, Switzerland). Microscopic analysis of the machined surface and chips was carried out using a workshop microscope (Advance ICD, Bresser, Rhede, Germany). Measurements of chip breaker profiles on the rake face were carried out using a Taylor Hobson profilometer (Leicester, UK) and Keyence 3D microscope (Osaka, Japan).

The experimental design plan was developed according to the Taguchi method [24]. The influence of the variable cutting data, i.e., feed, speed, and depth of cut (*f*, *v_c_*, *a_p_*), on the conditions of supplying cutting fluid with a high pressure value of 70 bar was analyzed. A constant value of the corner radius *r*_ε_ of the cutting insert was assumed −0.4 mm. In the statistical analysis of the test results, the model of the matching function according to the Formula (1) was adopted.

(1) $$Y_{1}=y-\varepsilon =b_{0}x_{0}+b_{1}x_{1}+b_{2}x_{2}+b_{3}x_{3}+b_{4}x_{4}$$

In the Formula (1) *Y*_1_ is the estimated response based on first order equation and *y* is the measured parameter (e.g., cutting force) on a logarithmic scale where *x*_0_ = 1 (dummy variable) and *x*_1_ − *x*_4_ are the logarithmic transformations of cutting speed, feed rate, and depth of cut, respectively; ε is the experimental error and ‘*b*’ values are the estimates of corresponding parameters.

The S/N (signal-to-noise) ratio analysis strategy was adopted as “the lowest-best” according to the Formula (2).

(2) $$S/N=-10\cdot \log\left(\frac{1}{n}\overset{n}{\underset{i=1}{\sum }}y_{i}^{2}\right)\;$$

where *y_i_* is the respective characteristic and *n* is the number of observations.

Table 2 presents the adopted ranges of the cutting data values. These values belong to the range recommended by the tool manufacturer for finishing machining. 

Table 3 presents the test plan together with the real values of the cutting data. 

## 3. Results

According to the adopted test plan, the components of the total cutting force were measured. The influence of the variable cutting parameters (*f*, *a_p_*, and *v_c_*) on the values of the total cutting force components, i.e., main *F_c_*, feed *F_f_*, and radial *F_p_*, was analyzed. Table 4 presents the obtained results of the S/N parameter and the average values of the individual components obtained in the individual tests system. Table 5, Table 6 and Table 7 present the statistical analysis of the test results (*DF*—degrees of freedom, *Seq SS*—sums of squares, *Adj SS*—adjusted sums of squares, and *Adj MS*—adjusted means squares). 

The analysis of the measurements results showed a linear dependence of the values of all components of the total cutting force the feed value *f* and the depth of cut *a_p_* when machining from the Grade 5 ELI titanium alloy in the HPC condition. The cutting speed has no significant effect on *F_f_* and *F_p_* component values of the total cutting force. Doubling of the cutting speed value resulted in a decrease in the value of the *Fc* component by approximately 10%. The results obtained are similar to those described in the literature. For example, Çolak [11] showed a lack or a minimum effect of liquid pressure on the change in the value of the components of the cutting forces. The values of the components of the cutting forces depend on the geometric dimensions of the cross-section of the cutting layer (i.e., the depth of cut and feed rate), the material properties, and the tool wear [16].

**Figure 1:**
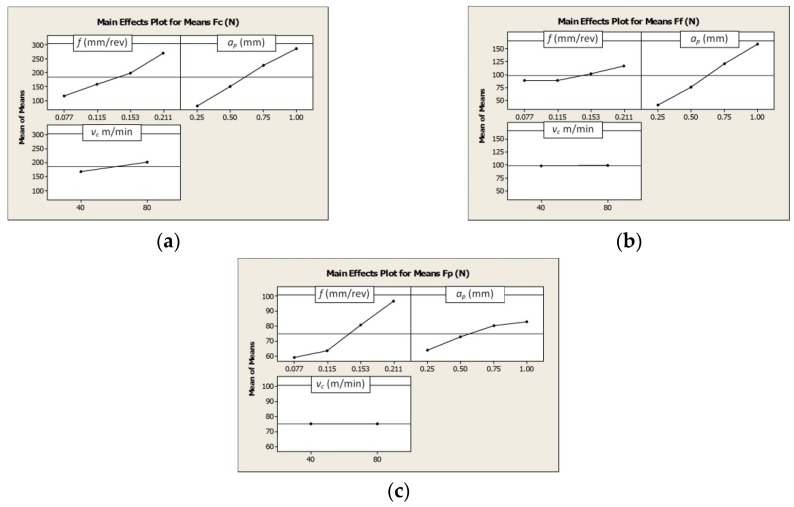
The influence of the cutting data on the values of the total cutting force components: (**a**) *F_c_*, (**b**) *F_f_,* and (**c**) *F_p_*.

Figure 1 shows graphically the influence of the particular cutting data on the values of the cutting force components.

Equations *F_c_*(*f*, *a_p_*, *v_c_*), *F_f_*(*f*, *a_p_*, *v_c_*), and *F_p_*(*f*, *a_p_*, *v_c_*) are described below as Equations (3)–(5), respectively.

(3) $$F_{c}\left(f,a_{p},v_{c}\right)=-197.84+1144.81f+279.52a_{p}+0.829v_{c},$$

(4) $$F_{f}\left(f,a_{p},v_{c}\right)=-36.33+231.41f+161.89a_{p}+0.0259v_{c},$$

(5) $$F_{p}\left(f,a_{p},v_{c}\right)=18.37+287.4f+26.12a_{p}-0.0162v_{c},$$

**Figure 2:**
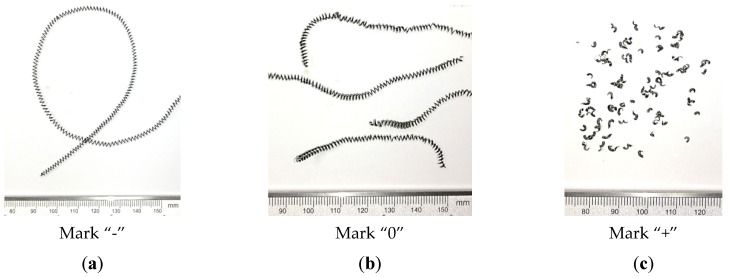
Sample photographs of the chips forms obtained in the cutting tests (**a**) *v_c_* = 80 m/min, *f* = 0.077 mm/rev, *a_p_* = 0.5 mm, (**b**) *v_c_* = 80 m/min, *f* = 0.115 mm/rev, *a_p_* = 0.5 mm, (**c**) *v_c_* = 80 m/min, *f* = 0.153 mm/rev, *a_p_* = 0.75 mm.

During the tests the analysis of the chip forms, their classification and evaluation were also carried out (Table 8). The longitudinal dimension of the chips was adopted as the main criterion when assessing the form of chips. A three-grade evaluation of the form of chips was adopted, i.e., preferred chips with a length of up to 20 mm, acceptable chips with a length of 20 to 100 mm, and unacceptable chips with a lengths exceeding 100 mm. The following marks were accepted when evaluating the chip form;”+”—chips correct; “−”—chips incorrect; and “0”—chips acceptable. Sample photographs of the chip shapes obtained during the cutting tests are shown in Figure 2.

Table 9 summarizes the evaluation of the chips form obtained in the experimental tests for finishing machining, i.e., in the range of the cutting depth *a_p_* = 0.25–1.0 mm and feed *f* = 0.077–0.211 mm/rev.

**Figure 3:**
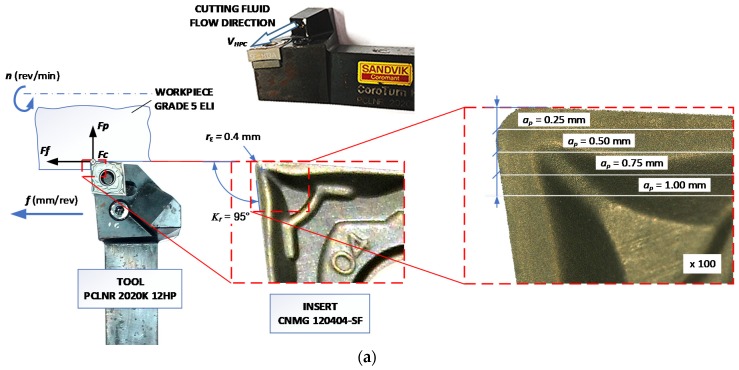

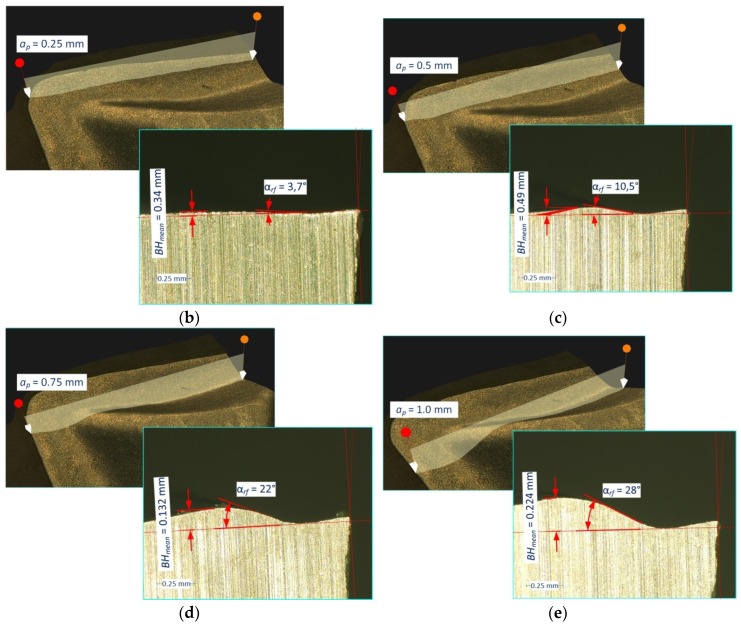
Scheme of the chip breaker profile measurements in the work system. (**a**) An example for the depth of cut *a_p_* = 0.5 mm, (**b**) *a_p_* = 0.25 mm, (**c**) *a_p_* = 0.5 mm, (**d**) *a_p_* = 0.75 mm, and (**e**) *a_p_* = 1.0 mm.

Analysis of the data obtained on this basis showed that for small cross-sections of the cutting layer *AD* < 0.06 mm^2^ and hence small cross-sections of chips the obtained form of chips is significantly influenced by the pressure of cutting fluid fed into the cutting zone. Chips produced in this range are preferred or accepted. For the increasing values of the cross-section of the cutting layer, the obtained form of chips changes to unacceptable. This results due to the increase of chips strength with a larger cross-section. For the cross-section *AD* > ~0.08–0.1 mm^2^, the chip form changes again and is advantageous (short arc chips). In this range of feeds and the depth of cut, the variable shape of the chip groove on the rake face (chip breaker) affects the change of the chips form. Figure 3 shows the measured profile of the -SF chip breaker in the tool layout (Figure 3a) at different cutting depths *a_p_*. Analysis of the obtained measurements showed significant increases in the angle of elevation α*_rf_* and the backwall height *BH* of the rear wall of the chip breaker to the depth of cut *a_p_* = 1.0 mm. The elevation angle of the rear wall of the breaker varies from 3.5° for the depth of cut *a_p_* = 0.25 mm to 27.3° for the depth of cut *a_p_* = 1.0 mm. The height of the rear wall of the chip breaker is characterized by a similar relationship. Changing the depth of cut from *a_p_* = 0.5 mm to *a_p_* = 1.0 mm causes an approximately threefold increase in the height of the backwall *BH*. 

**Figure 4:**
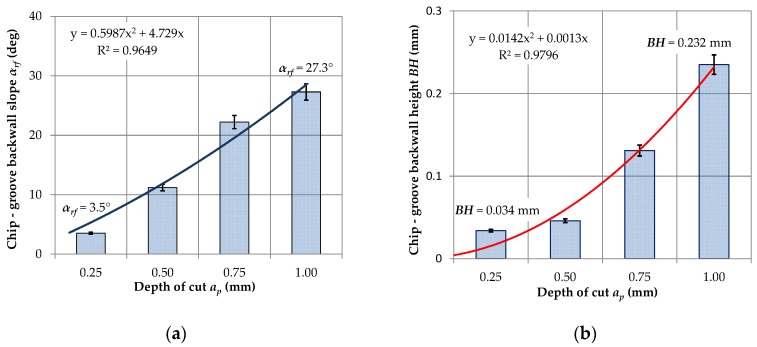
The dependence of the elevation angle of the rear wall of the breaker (**a**) and the height of the rear wall of the chip breaker (**b**) in the depth function.

Figure 4 shows the relationship between the angle of elevation *α_rf_* and the height of backwall of the chip breaker as a function of the depth of cut *a_p_*. The variable shape of the chip breaker significantly influences the change in chip forming. For small values of feed and depth of cut, there is no adequate filling of the chip groove with the workpiece material. The process of cracking and chip breaking depends on the value of cutting fluid pressure and the direction of its feeding into the cutting zone. Cutting fluid applied to the external surface of the chip causes increased chip rotation at a variable angle and its cracking. Chip breaking occurs in the thinnest places of the formed segments of the chip.

**Figure 5:**
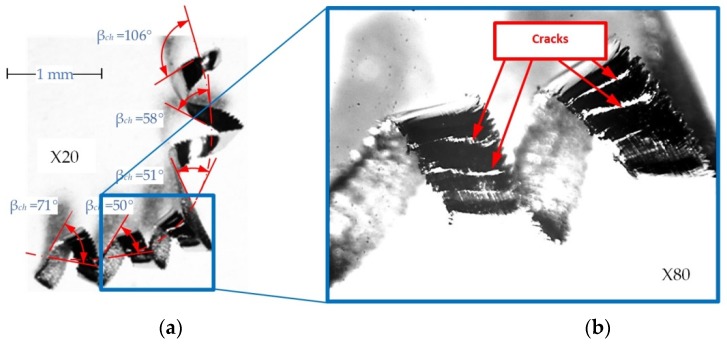
Microscopic photographs of chips (**a**) angle of curl—*f* = 0.077 mm/rev, *a_p_* = 0.25 mm and (**b**) external surface of the chip—*f* = 0.211 mm/rev, *a_p_* = 1.0 mm.

Figure 5a presents a photograph of chips with a selected angle of chip curl in the layout adopted by the authors, and Figure 5b shows examples of cracks on the outer surface of the chip. In order to determine the degree of filling of the chip groove and the method of chips forming, a turning process was simulated using the finite element method. 

Numerical simulations of the cutting process were performed according to a Lagrangian FE code [27]. Techniques such as adaptive remeshing and thermal analysis were integrated to model the complex interactions of the tool wedge and workpiece. The workpiece material was modeled as thermo-elastic-plastic, while the flow stress was considered to be a function of strain, strain rate, and temperature. The Johnson–Cook Equation (6) was used as the constitutive model. This approach is widely used because it is relatively simple (5 parameters) and numerically robust. Table 10 presents the main model parameters.

(6) $$\sigma \left(\alpha ,\;\dot{\alpha ,}T\right)=\left(A+B\alpha^{n}\right)\left(1+C\ln\left(\frac{\dot{\alpha }}{\dot{\alpha_{0}}}\right)\right)(1-{\left(\frac{T-T_{room}}{T_{melt}-T_{room}}\right)}^{m}$$

where, *A* is the yield stress, *B* is the strain hardening coefficient, *C* is the strain rate dependence coefficient, *n* is the strain hardening exponent, and *m* is the temperature dependence coefficient material parameters. *T_melt_* is the melting temperature for the material, $\dot{\alpha }$ is an equivalent plastic strain rate, and $\dot{\alpha_{0}}$ is the reference strain rate.

**Figure 6:**
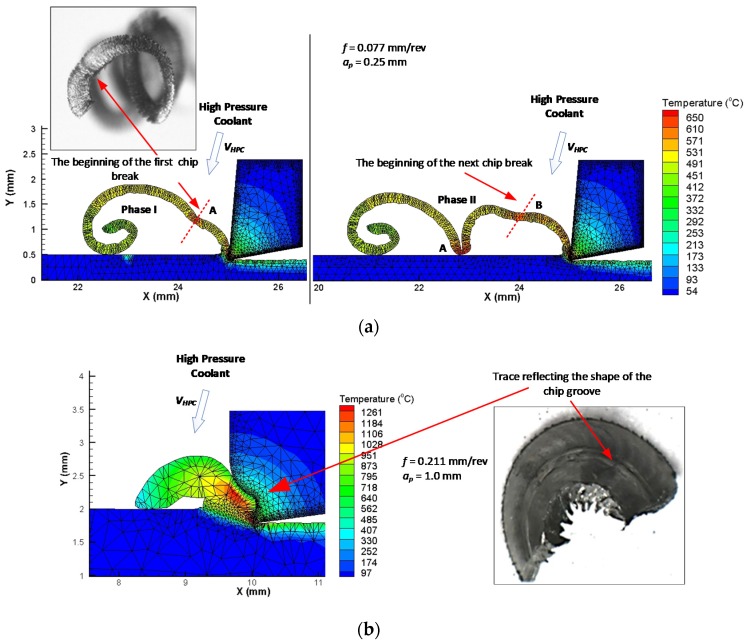
Examples of machining process simulations: (**a**) presentation of the stages of chip cracking process for feed *f* = 0.077 mm/rev, *a_p_* = 0.25 mm and view of the inner side of the chip and (**b**) presentation of chip groove filling for feed *f* = 0.211 mm/rev, *a_p_* = 1.0 mm and view of the outside of the chip.

In the simulation the cutting fluid pressure was 70 bar. The HPC has been modeled in the MES software as a boundary condition in the form of a coolant pressure acting directly on the chip. This is shown schematically using the vector *V_HPC_* in Figure 3a and Figure 6. Table 11 presents the main mechanical and thermal coefficients for the workpiece material.

The simulation of the cutting process, taking into account the variable shape of the chip breaker profile, showed that the most vulnerable area of the formed chip is along the line connecting particular chip segments. Figure 6a presents an example of the simulation result of the turning process for feed *f* = 0.077 mm/rev with selected chip segmentation areas. The cutting fluid pressure accelerates the cracking process in the thinnest place of the chip cross-section. 

Increases in the feed rate and depth of cut result in increases in the chip strength and the degree of filling of the chip groove. This also results in a change in the method of forming and breaking chips. The chip flowing on the surface of the rake largely fills the chip groove. In the initial forming phase, the chip rests on the rear wall of the chip breaker (Figure 6b). This contributes to the increase in the radius of curling the chip. In the next phase, the chip wraps and flows towards the cutting edge. The process of chip breaking occurs most often as a result of chip impact against the surface of the cutting tool. The cutting fluid pressure acting on the outside of the formed chip mainly supports the process of chip rolling. The simulation of the cutting process for the depth of cut *a_p_* = 1.0 mm and the feed *f* = 0.211 mm/rev showed that, for these parameters, almost complete filling of the chip groove occurs. This is confirmed by microscopic observations of the chip surface. Figure 6b shows a photograph of the outside of the chip surface. A longitudinal trace reflecting the shape of the chip groove is visible on the chip surface.

## 4. Conclusions

The aim of the presented research was to analyze machinability of the Grade 5 ELI titanium alloy with tools made of sintered carbides with defined chip breaker geometry in the condition of high pressure cutting fluid. The main aim of the analysis was to determine the areas of correct work of the chip breaker for the finishing turning process. The results of the analysis showed that

- the component values of the total cutting force depend on the feed rate and depth of cut and do not depend on the cutting speed;
- the obtained chip form (correct and unacceptable) depends on the range of feed rates and depth of cut, shape of the chip breaker and on the value of the cutting fluid pressure; and
- the curl and chip breaking process depend mainly on the feed rate and shape of the chip breaker.

The pressure direction and method of supplying the cutting fluid support the process of winding and chip breaking. For low feed values and cutting depths (low cross-section of the cutting layer and low mechanical strength of the chip) the pressure of the liquid delivered to the outside of the forming chip may initiate the cracking and chip breaking process. When increasing feed values, the chip strength increases and the cutting fluid cannot break the chip. In addition, there is an incomplete filling of the chip groove, which results in no chip strokes at the tool flank surface. In the range of high feed values there is a synergistic effect of the filling of the chip groove and the pressure of the cutting fluid supporting the process of chip rolling. The cutting liquid pressure value supports chip breaking by changing the direction of the chip flow. This results in a correct chip breaking cycle. 

## Figures and Tables

**Table 1:** Chemical composition—Grade 5 ELI (Extra Low Interstitials) (%).

	Al	V	Fe	C	N	O	H	Ti
Result	6.10	4.13	0.05	<0.01	0.01	0.10	0.003	Remainder

**Table 2:** The variables values in the research plan.

Trial	Coded Parameter	Real Parameter	min	Value		max
1.	A	*f* (mm/rev)	0.077	0.115	0.153	0.211
2.	B	*a_p_* (mm)	0.25	0.50	0.75	1.00
3.	C	*v_c_* (m/min)	40			80

**Table 3:** Research plan with real values.

Trial	A	B	C	*f* (mm/rev)	*a_p_* (mm)	*v_c_* (m/min)
1.	1	1	1	0.077	0.25	40
2.	1	2	1	0.077	0.50	40
3.	1	3	2	0.077	0.75	80
4.	1	4	2	0.077	1.00	80
5.	2	1	1	0.115	0.25	40
6.	2	2	1	0.115	0.50	40
7.	2	3	2	0.115	0.75	80
8.	2	4	2	0.115	1.00	80
9.	3	1	2	0.153	0.25	80
10.	3	2	2	0.153	0.50	80
11.	3	3	1	0.153	0.75	40
12.	3	4	1	0.153	1.00	40
13.	4	1	2	0.211	0.25	80
14.	4	2	2	0.211	0.50	80
15.	4	3	1	0.211	0.75	40
16.	4	4	1	0.211	1.00	40

**Table 4:** Test results for cutting forces measurements *F_c_*, *F_f_*, *F_p_*.

Number	A	B	C	*f* (mm/rev)	*a_p_* (mm)	*v_c_* (m/min)	S/N *F_c_*	*F_c_mean_* (N)	S/N *F_f_*	*F_f_mean_* (N)	S/N *F_p_*	*F_p_mean_* (N)
1.	1	1	1	0.077	0.25	40	−34.4	52.5	−34.4	52.5	−34.4	52.5
2.	1	2	1	0.077	0.50	40	−39.4	92.7	−35.9	62.4	−35.9	62.4
3.	1	3	2	0.077	0.75	80	−43.0	141.5	−35.7	61.1	−35.7	61.1
4.	1	4	2	0.077	1.00	80	−45.1	180.2	−35.6	59.8	−35.6	59.8
5.	2	1	1	0.115	0.25	40	−36.7	68.5	−35.5	59.6	−35.5	59.6
6.	2	2	1	0.115	0.50	40	−41.6	119.9	−32.8	43.4	−32.8	43.4
7.	2	3	2	0.115	0.75	80	−45.8	193.6	−37.7	76.1	−37.7	76.1
8.	2	4	2	0.115	1.00	80	−47.9	248.5	−37.4	74.2	−37.4	74.2
9.	3	1	2	0.153	0.25	80	−38.9	88.1	−37.0	71.1	−37.0	71.1
10.	3	2	2	0.153	0.50	80	−44.7	171.5	−38.7	86.0	−38.7	86.0
11.	3	3	1	0.153	0.75	40	−47.3	231.5	−38.3	81.8	−38.3	81.8
12.	3	4	1	0.153	1.00	40	−49.5	299.0	−38.5	84.1	−38.5	84.1
13.	4	1	2	0.211	0.25	80	−40.9	110.3	−37.2	72.6	−37.2	72.6
14.	4	2	2	0.211	0.50	80	−46.7	216.2	−39.9	99.2	−39.9	99.2
15.	4	3	1	0.211	0.75	40	−50.6	338.3	−40.2	102.3	−40.2	102.3
16.	4	4	1	0.211	1.00	40	−52.4	417.6	−41.1	113.3	−41.1	113.3

**Table 5:** Analysis of variance for average value *F_c_*.

Source	DF	Seq SS	Adj SS	Adj MS	F	*p*
A	3	51,562	51,562	17,187.4	24.15	0.000
B	3	96,989	96,989	32,329.5	45.43	0.000
C	1	4561	4561	4560.8	6.41	0.035
Residual Error	8	5693	5693	711.6		
Total	15	158,804				

**Table 6:** Analysis of variance for average value *F_f_*.

Source	DF	Seq SS	Adj SS	Adj MS	F	*p*
A	3	2187.8	2187.8	729.3	2.00	0.193
B	3	32,350.1	32,350.1	10,783.4	29.54	0.000
C	1	5.4	5.4	5.4	0.01	0.906
Residual Error	8	2920.3	2920.3	365.0		
Total	15	37,463.7				

**Table 7:** Analysis of variance for average value *F_p_*.

Source	DF	Seq SS	Adj SS	Adj MS	F	*p*
A	3	3613.10	3613.10	1204.37	10.61	0.004
B	3	866.17	866.17	288.72	2.54	0.130
C	1	0.03	0.03	0.03	0.00	0.987
Residual Error	8	908.25	908.25	113.53		
Total	15	5387.55				

**Table 8:** Chips forms obtained in the cutting tests.

		*f* = 0.077 mm/rev	*f* = 0.115 mm/rev	*f* = 0.153 mm/rev	*f* = 0.211 mm/rev
	
*a_p_* = 0.25 mm		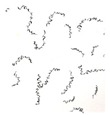	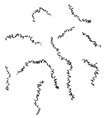	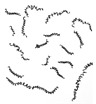	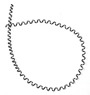
*a_p_* = 0.50 mm		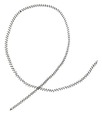	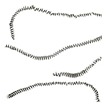	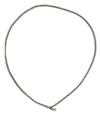	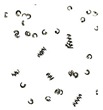
*a_p_* = 0.75 mm		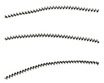	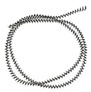	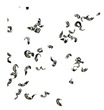	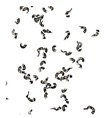
*a_p_* = 1.00 mm		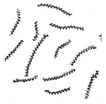	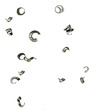	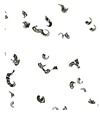	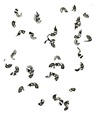

**Table 9:** Work areas of the SM chip breaker and the estimation of the chips’ form.

		*f* = 0.077 mm/rev	*A_D_* (mm^2^)	*f* = 0.115 mm/rev	*A_D_* (mm^2^)	*f* = 0.153 mm/rev	*A_D_* (mm^2^)	*f* = 0.211 mm/rev	*A_D_* (mm^2^)
	
*a_p_* = 0.25 mm		+	0.02	0	0.03	+	0.04	−	0.05
*a_p_* = 0.50 mm		−	0.04	0	0.06	−	0.08	+	0.11
*a_p_* = 0.75 mm		−	0.06	−	0.09	+	0.11	+	0.16
*a_p_* = 1.00 mm		0	0.08	+	0.12	+	0.15	+	0.21

**Table 10:** Material constants of the J–C flow stress model for titanium alloy [28,29].

*A* (MPa)	*B* (MPa)	*n*	*C*	*m*	*T_melt_* (°C)
997.9	653.1	0.45	0.0198	0.7	1277

**Table 11:** Mechanical and thermal coefficients used in simulation.

Thermal Conductivity (W/m·degC)	Heat Capacity (J/Kg·degC)	Density (Kg/m^3^)	Young’s Modulus (Pa)	Poisson’s Ratio
6.6	526	4430	1.1 × 10^11^	0.31

## Nomenclature

*f*	Feed (mm/rev)
*a_p_*	Depth of cut (mm)
*r_ɛ_*	Nose radius (mm)
*v_c_*	Cutting speed (m/min)
*D_C_*	Workpiece diameter (mm)
*Ra*	The average arithmetic deviation of the profile from the mean line (µm)
*F_c_*	Main component of the total cutting force (N)
*F_f_*	Feed component of the total cutting force (N)
*F_p_*	Radial component of the total cutting force (N)
*α_rf_*	Chip—groove backwall slope (deg.)
*BH*	Chip—groove backwall height (mm)
*W_cfc_*	Coefficient of chip classification
*A_D_*	Cross-sectional area of cut (mm^2^)
β*_ch_*	Angle of chip twist (deg.)
